# Bladder Sparing Approaches for Muscle-Invasive Bladder Cancers

**DOI:** 10.1007/s11864-016-0390-8

**Published:** 2016-03-04

**Authors:** Omar M. S. El-Taji, Sameer Alam, Syed A. Hussain

**Affiliations:** Department of Surgery, St. Helen’s and Knowsley NHS Teaching Hospitals, University of Liverpool, Prescot, L35 5DR UK; Department of Molecular and Clinical Cancer Medicine, University of Liverpool, Liverpool, L69 3GA UK

**Keywords:** Bladder cancer, Muscle-invasive, Bladder sparing, Organ preservation, Bladder preservation, Chemoradiation

## Abstract

Organ preservation has been increasingly utilised in the management of muscle-invasive bladder cancer. Multiple bladder preservation options exist, although the approach of maximal TURBT performed along with chemoradiation is the most favoured. Phase III trials have shown superiority of chemoradiotherapy compared to radiotherapy alone. Concurrent chemoradiotherapy gives local control outcomes comparable to those of radical surgery, but seemingly more superior when considering quality of life. Bladder-preserving techniques represent an alternative for patients who are unfit for cystectomy or decline major surgical intervention; however, these patients will need lifelong rigorous surveillance. It is important to emphasise to the patients opting for organ preservation the need for lifelong bladder surveillance as risk of recurrence remains even years after radical chemoradiotherapy treatment. No randomised control trials have yet directly compared radical cystectomy with bladder-preserving chemoradiation, leaving the age-old question of superiority of one modality over another unanswered. Radical cystectomy and chemoradiation, however, must be seen as complimentary treatments rather than competing treatments. Meticulous patient selection is vital in treatment modality selection with the success of recent trials within the field of bladder preservation only being possible through this application of meticulous selection criteria compared to previous decades. A multidisciplinary approach with radiation oncologists, medical oncologists, and urologists is needed to closely monitor patients who undergo bladder preservation in order to optimise outcomes.

## Introduction

Bladder tumours are the 2nd most common genitourinary tumours and the 9th most common cause of cancer-associated morbidity worldwide [1]. In the USA for 2015, it is estimated that 74,000 new cases of bladder cancer (BC) will be diagnosed, and there will be an estimated 16,000 cases of BC-associated mortality [2]. It signifies the most common urinary tract malignancy, with a metastatic bladder cancer median survival rate that seldom exceeds 15 months. A changing age distribution of western societies and a progressing bequest of cigarette smoking have meant that bladder-related cancers have accelerated, becoming a significant cause of death [3].

**Fig. 1:**
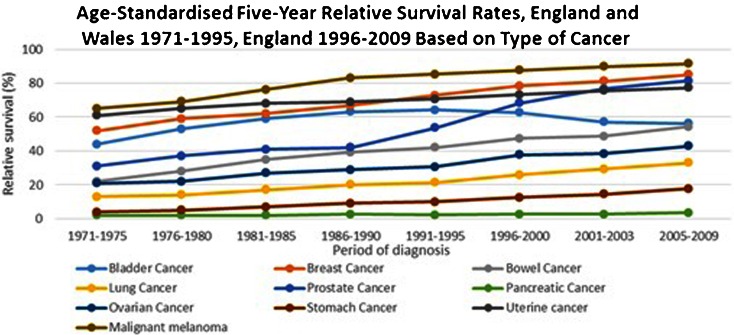
Age-standardized 5-year relative survival rates, England and Wales 1971–1995, England 1996–2009 based on type of cancer

When comparing the 5-year relative survival rates of the most common UK malignancies from 1970 to 2009, all malignancies show a rise in relative survival across the years, apart from BC which shows a 12.5 % reduction in relative survival from 1991 to 2009 (Fig. 1) [4]. The management of muscle-invasive bladder cancer (MIBC) remains a multifactorial challenge as well as the eradication of local disease, the eradication of micrometastasis, and finally the maintenance of an optimal quality of life.

Considering that the mean age at diagnosis is 70, and the single most significant risk factor is smoking, many patients present with existing co-morbidities, rendering certain interventions highly risky and therefore presenting limited options for treatment. At initial presentation, 30 % of patients are diagnosed with a muscle-invasive tumour. From the remainder of patients that had been diagnosed with a non-muscle invasive tumour at presentation, 30 % go on to develop muscle-invasive disease during follow-up [5]. With the highest predisposition to relapse of any malignancy, patients with BC require regular surveillance and close follow-up during and, more significantly, in succeeding therapy [6]. The management of bladder tumours has remained largely unchanged over the last few decades. The assumed gold standard treatment for organ-confined MIBC is radical cystectomy (RC), with bilateral dissection of pelvic lymph nodes (PLND). Although RC aims to be curative, at least half of patients with muscle-invasive disease develop metastasis within a 2-year period and subsequently die [7]. Furthermore, patients undergoing RC will often be left with a replacement voiding system which uses autologous segments of gastrointestinal tissue [8]. This is often achieved through ileal conduit urinary diversion, orthotopic bladder substitution or continent cutaneous diversion [9, 10]. Whilst radical extirpation of the bladder can be successful, from an oncological perspective, substantial morbidity is associated with enteric interposition of gastrointestinal tissue within the urinary tract [11, 12]. Surgical intervention itself requires procedures on the urinary and gastrointestinal tracts which are associated with complication rates of approximately 61 % [13]. The average post-operative length of stay ranges from 7 to 14 days with a post-operative 90-day readmission rate of 32 % as demonstrated by Aghazadeh et al. [14–16]. In a similar study, Goodney et al. reported a 30-day readmission rate of 21 %, the second highest rate of any surgical procedure, only surpassed by mitral valve replacement (22 %) [16].

Despite continent reconstruction of the urinary tract using cutaneous reservoirs or orthotopic diversions being a stepup from ileal conduit urinary diversion, a limited number of patients in practice will have continent urinary reconstruction [17]. Moreover, whilst the use of tissue engineering in the field of bladder reconstruction post-cystectomy shows great potential, its clinical application in mainstream urological practice seems still quite a way off [18]. The use of bladder-sparing approaches with regards to MIBC has been extensively investigated over the past two decades. These approaches have been developed to address the needs of two main cohorts: patients with severe medical co-morbidities for whom a radical surgery is too high risk and patients with limited disease who wish to avoid radical surgery.

The general consensus is that single-modality bladder-preserving approaches consisting of chemotherapy, radiotherapy or transurethral resection of bladder tumour (TURBT) are inferior and often yield inferior results for local tumour control and long-term survival in MIBC. Consequently, dual/trimodality regimens have been used to attempt to replicate the outcomes of RC, whilst avoiding the morbidity and impact on quality of life associated with radical therapy. The trimodality approach involves an extensive TURBT, attempting to achieve complete resection. Complete resection may not always be possible, and incomplete resection is associated with a reduced disease-free survival [19]. Induction therapy then follows with surveillance cystoscopy and biopsy to assess clinical response. Complete responders will progress to chemoradiation whilst non-responders will undergo cystectomy.

Historically, bladder-sparing approaches were initially used in patients who were not candidates for surgery; however, despite this poorly selected population, the 5-year local control rate for these patients was 20–40 % whilst overall survival (OS) was 50 % [20–23]. These results were always seen to be inferior to surgery, and therefore their role in the immediate management of BC was rarely explored in a more selective cohort of patients.

Phase I and II trials in the 1980s showed that metastatic BCs are chemosensitive, fostering hope of cure. Various treatment algorithms have been proposed to aid in patient selection for bladder preservation and their risk stratification towards appropriate treatment options—no doubt the most vital factors in BC management [24]. Although accumulated data over the last 20 years has suggested that concomitant chemoradiation in MIBC gives equivalent results to those of RC, it has never been formally compared in a randomised control trial.

The difficulty in achieving both complete treatment response whilst ensuring bladder function remains acceptable and has driven urologists and oncologists to work together to develop multimodal approaches to MIBC. Here, we present a review of the current literature related to the bladder sparing approaches for MIBC.

### Radical TUR

TUR involves the transmural resection of the bladder wall and resecting all visible tumours as far out as the perivesical connective tissue [25]. This extensive resection comes with significant risks including bladder perforation and increased risk of tumour dissemination through extravesical seeding [26].

Primarily, the rationale behind radical TUR as the only treatment is the discovery of pT0 at RC in those previously only treated with TUR before surgery. Studies have shown a >10 % incidence of pT0 at RC [27–30]. Furthermore, 42 % of those with cT2a disease have been shown to have pT0 disease after visibly completing TUR [30]. Even those with pT0 are not all truly free of disease as recurrence-free survival (RFS) has been reported as 92 and 86 % at 5 and 10 years, respectively [28].

The overall consensus amongst the majority of experts is that TUR alone is inadequate for a diagnosis of MIBC. The reasons behind this are extensive; however, one factor deemed significant is the high rate of upstaging at RC. Studies have demonstrated upstaging from cT2 to pT3 as high as 39.7 % [31] with lymph node metastasis at the time of cystectomy at 23 % as reported in several series [28, 31, 32]. Patient selection is therefore vital and is often used as a last resort for those unwilling or unfit to undergo more aggressive treatment. Although limited data exists for long-term outcomes of radical TUR, two prospective studies have been the basis of radical TUR monotherapy with distinctive results (Table 1).

**Table 1:** Radical TUR monotherapy

					Overall disease-specific survival (%)				Ref
Study	Study design	Study group	Exclusion criteria	Bladder preservation cohort	Follow-up	5 years	10 years	15 years	
Herr et al.	Phase II nonrandomized trial	TUR	No tumour/residual CIS/NMIBC on repeat TUR	99	≥10 years	82	76		[33]
Solsona et al.	Phase II nonrandomized trial	TUR	Nodular tumour of >3 cm or those with evidence of advanced clinical stage disease (hydronephrosis/adenopathy/metastasis)	133	15 years	82	80	77	[34]

It is vital to adopt appropriate and accurate patient selection when choosing candidates which may have successful outcomes from radical TUR as detailed in the above studies. Ideal candidates boasted no evidence of advanced clinical stage disease, tumours <3 cm, and negative bed biopsies at initial TUR or negative restaging TUR [33–35].

Surveillance and rigorous follow-up similar to that in the study by Solsona et al. may not be feasible. Monotherapy showed inadequacies in patients who developed disease reoccurrence, and, therefore, for these patients more aggressive treatment with multimodal approaches could be beneficial. These patients eventually required RC or further treatment for residual disease/local relapse [33, 34]. BCs are known to have the highest susceptibility of recurrence and progression of any malignancy. With such high odds of recurrence, BC has produced the most expensive management protocol of any malignancy, costing the National Health Service approximately £55.39 million in the UK per annum [6].

### Radiotherapy

Conventionally, radiotherapy is administered externally in a typical dose schedule of 64 Gy in 32 fractions or hypofractionated schedules such as 55 Gy in 20 fractions, both shown to be equally as effective in the long term [36••]. Although a clear dose-response relationship has not been identified, studies including a multivariant analysis have shown poorer outcomes when using doses less than 57.5 Gy [37, 38]. It is important, however, to appreciate that increasing dosages of radiotherapy is closely linked with toxicity, but the majority of modern regimens are tolerated well and long-term bladder function is seldom effected [39]. Despite its merits, however, its use has declined markedly since the 1980s and radiotherapy alone is now used only in circumstances where other treatment modalities have failed, or in advanced disease. It is important to note that radiotherapy is seldom used alone and that the majority of patients have often already undergone prior TUR.

Although surgery is considered the standard of care in the majority of patients, considerable interest in bladder preservation has led to radiotherapy re-emerging as an effective alternative, particularly in patients who are not fit for radical surgery. Due to the cohorts that are treated with radiotherapy, it is often difficult to compare outcomes of radiotherapy with surgery. Patients undergoing radical radiotherapy in comparison with surgical candidates often have multiple co-morbidities with worse prognostic factors and poorer performance statuses [40, 41]. It is for these reasons that studies have reported relatively high rates of incomplete response and local recurrences that reach up to 50 % [42].

Studies have demonstrated outcomes that remain inferior to multimodal treatment modalities, with a 5-year overall survival (OS) of 26–50 % and local control rates of approximately 50 % [22, 43–49].

Pollack et al. assessed response to radiotherapy in different tumour stages and reported a 5-year OS for T2 to be 40–59 % and 0–50 % for T3 [45]. In a similar study, Pollack et al. reported local long-term control rates after radiotherapy alone in T3b at 27 % [20]. Factors that have been found to be statistically significant in prognosticating local failure are tumour multiplicity, ureteric obstruction and higher T stage [22].

Shipley et al. evaluated data from 55 patients treated with radiotherapy alone in order to identify factors associated with tumour radio responsiveness. Sixty-seven percent of patients presented with T2 or T3 tumours with the remainder of patients having T4 tumours. OS after 5 years was 28 %; however, when comparing patients who possessed T2/3 with those having T4 disease, survival after 5 years was 45 and 9 %, respectively. The T2/3 group demonstrated that the most vital prognostic factor was histological finding of tumour on papillary surface, in the absence of urethral obstruction, and in less advanced clinical stages, factors that were similarly found by Pollack et al. [19, 45].

It is important to mention, however, that both population-based studies and large surgical and radiotherapy-based series have shown similar long-term survival rates and no survival difference linked to mode of treatment [28, 48, 50]. Additionally, the vast majority of these large-scale surgical series have population medians which are well below the disease population, signifying its lack of application in patients with invasive bladder disease [28]. In a large population-based series, Munro et al. investigated the outcomes of 458 patients with MIBC undergoing RC versus radiotherapy. OS was similar in the two groups, 22 % for those undergoing radiotherapy, and 24 % in the group undergoing RC [51].

Although chemoradiotherapy has much better outcomes as a treatment option in comparison with radiotherapy alone, a small proportion of patients will not be candidates for dual therapy and radiotherapy alone may be the only treatment option available. A highly conclusive study by James et al. demonstrated that chemoradiotherapy significantly improved loco-regional control of BC with no significant increase in adverse events compared with radiotherapy alone. They also provided evidence, which would suggest that RT alone does have at least some curative potential. Loco-regional DFS at 2 and 5 years was 54 and 35 %, respectively [36••].

### Chemotherapy

Chemotherapy has been used for decades in the management of MIBC as neoadjuvant or adjuvant adjuncts to primary RC or as an organ preservation treatment. This has shown to reduce the risk of micrometastasis, improving survival [52, 53]. A recent international multicentre-randomised phase III trial investigated the use of neoadjuvant cisplatin, methotrexate and vinblastine (CMV) chemotherapy in patients with MIBC treated by cystectomy and/or radiotherapy [54]. This trial demonstrated long-term statistically significant reduction in risk of death and a 6 % increase in a 10-year survival after CMV therapy, compared with radiotherapy or RC alone. The addition of paclitaxel to GC demonstrated superiority through a higher response rate as well as survival benefit which did not reach statistical significance [55]. Chemotherapy alone does not play any role in the management of MIBC.

### Synchronous Chemoradiation

The first bladder preservation studies were conducted in the early 1980s and were found to demonstrate encouraging results [47, 48, 56–59]. Rationale that supports the addition of chemotherapy to radiotherapy is based on four main principals. Firstly, the evidence of micrometastasis exists concurrently with invasive BC. In a study of 367 individuals at autopsy, 68 % demonstrated distant metastasis, with frequency of metastasis increasing with tumour stage as one would expect (pT2 36 %, pT3a 45 %, pT3b 69 % and pT4 79 %) [60].This is supported by the high probability of consequent distant metastasis post-cystectomy or radiotherapy alone by approximately 50 % after 2 years [28]. This suggests that combination therapy would reduce the likelihood of distant metastasis by tackling micrometastatic disease. Secondly, the use of radiotherapy on its own can cause the development of vascular sclerosis after a period of months, thus increasing the likelihood of chemotherapeutic failure [61]. Thirdly, the prime time to tackle potential micrometastatic cells is prior to the development of gross metastatic disease. Finally are the radiosensitising properties of certain chemotherapeutic agents, e.g., gemcitabine, mitomycin C and 5-fluorouracil [62].

Although a variety of phase I and II trials have demonstrated on the whole good tolerability and feasibility when assessing chemoradiation, data is still lacking with very few trials that compare synchronous chemoradiation with radiation alone [42, 48, 63–74]. In fact, the only randomised trial of this approach before BC2001 [36••] and BCON [75••] was that in 1996 by the Canadian Cancer Society Research Institute [57]. This study randomised 99 patients to receive radiotherapy of 40 Gy in 20 fractions alone or radiotherapy with the same schedule combined with cisplatinum 100 mg/m^2^ twice weekly for 3 weeks, followed by elective cystectomy or further radiotherapy. Those who had received combined therapy had a significantly lower rate of loco-regional recurrence compared to those receiving radiotherapy alone (40 vs 59 % *p* = 0.026). Statistically, no significant results were obtained for bladder preservation (70 % vs 36 % *p* = 0.14) and OS (47 % vs 33 % p = 0.34). Although this is a small study with limited power, the promising results justified further investigation.

Many studies have evaluated the efficacy of synchronous chemoradiation compared to radiotherapy alone in tumours arising from primary sites other than the bladder [76–78]. These studies suggested a substantial advantage in local control and reduction in odds of death with combined therapy. One such study is the UKCCCR, which compared radiotherapy with synchronous chemotherapy in anal cancer [78]. Here, patients were randomised to receive radiotherapy alone or radiotherapy with 5-fluorouracil (5FU) by continuous infusion during the first and last weeks of radiotherapy in addition to mitomycin C on day 1 of the first fraction of radiotherapy. The significance of this study in the management of BC came about because of the significant prevalence of renal impairment amongst the vast majority of BC patients. Cisplatin itself requires an adequate GFR (>60 mL/min) for it to be safely delivered and to justify its side effect profile [79]. When comparing the dosing regimen from the Canadian study to our institution, the vast majority of patients would be deemed unfit to receive cisplatin at those doses. Our previous work has demonstrated in a phase I trial that radiotherapy and concurrent chemotherapy with mitomycin C and 5FU are feasible with acceptable toxicity in patients with poor prognosis [73]. Hussain et al. then subsequently conducted a phase II trial investigating synchronous chemoradiotherapy with mitomycin C and infusional 5FU in MIBC. We reported the long-term toxicity and efficacy results with the optimised regimen. Patients were administered mitomycin and 5FU for 5 days during weeks 1 and 4 of radiotherapy (55 Gy in 20 fractions). OS was 68 % (12 months), 49 % (36 months) and 36 % (60 months). It is worth noting that many patients entering our phase I/II study were deemed unfit for radical cystectomy. Local and distant progression free rates were 82 and 86 % at 12 and 24 months, respectively, and 79 and 75 % at 24 months. We concluded that using multimodality therapy is feasible and safe in patients with poor renal function [80]. Choudhury et al. demonstrated in a phase II trial a high response rate, durable local control, and acceptable toxicity in patients treated with concurrent chemoradiotherapy using gemcitabine [74].

James et al., following on from the phase I/II study, demonstrated in a phase III randomised control trial comparing TURBT with either synchronous chemoradiation (mitomycin C and 5FU) or with radiotherapy alone (either 55 Gy in 20 fractions over 4 weeks or 64 Gy in 32 fractions over 6.5 weeks) that chemoradiation significantly improved loco-regional control of BC with no significant increase in adverse events compared with radiotherapy alone [36••]. This study with a median follow-up of 69.9 months demonstrated a relative reduction in loco-regional recurrence of 35 % and almost halving of invasive recurrence. At 2 years, loco-regional DFS was more superior in the chemoradiation arm compared to radiotherapy alone (67 vs 54 %, respectively). The 5-year OS rate was reported to be 48 % in the chemoradiation arm compared to 35 % in the radiotherapy group (HR, 0.82; 95 % CI 0.63–1.09; *p* = 0.16). Acute toxic effects were also shown not to reach statistical significance in relation to grades 3 and 4 outcomes (27.5 % vs 365, *p* = 0.07). Bladder volume irradiated did however correspond to risk of grade 3 or 4 toxicity. Bladder function, an important contributor to quality of life, was demonstrated not to be significantly more impaired in the chemoradiotherapy arm. Good long-term bladder function and low rates of salvage cystectomy suggest that the use of chemoradiotherapy in BC patients who are often frail, with multiple co-morbidities, appears to be of significant benefit.

Hoskin et al. compared 333 patients with locally advanced BC in a phase III trial receiving either radiotherapy alone (schedule of either 55 Gy in 20 fractions in 4 weeks or 64 Gy in 32 fractions in 6.5 weeks) or synchronous chemoradiation (carbogen and nicotinamide). Chemoradiotherapy showed a non-significant improvement in the primary end point (cystoscopic control at 6 months) compared to radiotherapy alone (81 vs 76 %, *p* = 0.3) [75••]. Differences in 3-year OS (59 vs 46 %, *p* = 0.05), risk of death (14 % lower, *p* = 0.04) and local relapse (*p* = 0.05) were significantly in favour of chemoradiotherapy. There was no difference in acute bowel and urinary events between the two groups. Late events only show an increase in morbidity of grade 2 or worse stool frequency in the treatment group. This randomised control trial showed that the use of radiotherapy with concurrent chemotherapy using nicotinamide and carbogen demonstrates a schedule which has acceptable levels of toxicity, comparable with what is encountered with radiotherapy alone. Some therapeutic gain can be concluded from this study, although non-significant improvements in loco-regional control may suggest the need for further radiosensitizers, e.g., cisplatin, different to that of the hypoxic modification mechanism of nicotinamide and carbogen.

Taken together, trial data with chemoradiation suggests tolerability in those with poorer renal function and other existing co-morbidities whether treated with the North American trimodality approach [72] or the UK single treatment block [36••]. Other phase III studies have also demonstrated encouraging results (Table 2).

**Table 2:** Phase III chemoradiation trials

Study	n	Tumour grade	Methods	Complete response rate	Results	Ref
Hoskin et al.	333	T2–T4a	Radiotherapy (55 Gy in 20 fractions in 4 weeks or 64 Gy in 32 fractions in 6.5 weeks) + nicotinamide (60 or 40 mg/kg given 1.5–2 h before each fraction) + carbogen (2 % CO_2_ and 98 % O_2_ at 15 L/min administered 5 min before and during radiotherapy) vs radiotherapy alone	81 vs 76 % (*p* = 0.3)	3-year overall survival 59 vs 46 % (*p* = 0.04)	[75••]
Coppin et al.	99	T2–T4b N0	Radiotherapy (fractionated continuous pelvic radiation) + cisplatin 100 mg/m^2^ at 2-week intervals for three cycles concurrent with radiation vs radiotherapy alone	47 vs 31 %	3-year overall survival 47 vs 33 % 5-year locoregional relapse rate 40 vs 59 % (*p* = 0.04) bladder preservation 70 vs 36 %	[57]
James et al.	360	T2–T4a N0	Concominant chemoradiotherapy (5FU 500 mg/m^2^/day during fractions 1–5 and 16–20 and mitomycin C 12 mg/m^2^ + whole bladder radiotherapy/modified volume radiotherapy) vs radiotherapy alone		5-year overall survival 48 vs 35 % (*p* = 0.16) 2-year disease-specific survival 67 vs 54 % (*p* = 0.03)	[36••]
Tunio et al.	230	T2–T4 N0/Nx	Concomitant chemoradiotherapy with whole pelvis radiotherapy vs bladder-only radiotherapy	93 vs 96 % (*p* = 0.05)	5-year overall survival 52.9 vs 51 % (*p* = 0.8) 5-year disease-specific survival 47.1 vs 46.9 % (*p* = 0.5) Bladder preservation rates 58.9 vs 57.1 % (*p* =0.8)	[81•]

### Quality of Life and Toxicity of Organ Preservation

It is important to mention that perioperative morbidity and mortality of salvage cystectomy after previous bladder chemoradiation is very similar to that of primary cystectomy [82••]. Recent studies assessing bladder function in trimodality approaches have demonstrated in 71 patients (median follow-up 6.3 years) that 75 % had normally functioning bladders based on urodynamic studies and 85 % reported no urgency [83]. It is important to comment that quality of life and treatment toxicity depend on treatment regimens. Patient preference and likelihood of treatment success should drive modality selection. It is however important to consider the benefit of organ preservation over radial cystectomy. Several quality of life studies have evaluated the reconstructive methods of RC. A Japanese study assessed 85 patients post-reconstructive cystectomy (orthotopic bladder or ileal conduit) [84]. No significant difference was found between the modality of reconstruction; however, when compared with the general population in the USA, general health and social functioning was significantly lower. Another major concern following RC is erectile dysfunction; studies have shown an overall increase in erectile dysfunction, even in nerve-sparing surgery [85, 86].

### Future Directions

In an effort to improve OS and better control of loco-regional disease, novel approaches have been trialed. The use of hyperfractionated radiotherapy is one such approach. The RTOG protocol 07–12 studied the use of hyperfractionated radiotherapy (twice daily) with concurrent cisplatin therapy which achieved a complete response of >85 % [87]. Other studies, however, comparing chemoradiation with radiation alone using two fractionation schemes have demonstrated no difference for loco-regional DFS [36••]. Another novel approach to radiotherapy that has been used is image-guided radiation therapy (IGRT): this conformal radiotherapy more accurately targets tumours in mobile organs like the bladder [88]. Here, three to four gold markers are implanted into the bladder during TURBT and then tracked during radiotherapy. This allows the radiographer to ensure the tumour is within the desired radiotherapy field. However, these plates can often cause pain or fall out altogether [89]. An alternative to this approach is using the contrast agent lipodol. This works in the same way as plate insertion in that during TURBT, lipodol is injected into the tumour sites and radiotherapy can then focus its field of treatment. This use of IGRT is ideal as it can dictate everyday radiotherapy based on the location of the tumour within the bladder [89]. Bladders will change in shape and size depending on bowel distention and the volume of urine in the bladder. Some agents are currently being investigated for use alongside radiotherapy. Chakravarti et al. demonstrated that there is significant correlation between reduced response rate and HER2/neu overexpression [90]. HER2/neu positivity can be found in 40–80 % of bladder tumours. Hussain et al. reported in a multicenter phase II trial using trastuzumab, carboplatin, gemcitabine and paclitaxel in advanced metastatic urothelial carcinoma patients, with a median time to progression and survival of 9.3 and 14.1 months, respectively [91]. This study demonstrated feasibility; however, toxicity rates, especially cardiac toxicity, were high. Theoretically, there may therefore be a role of trastuzumab therapy in HER2/neu-positive bladder tumours in combination with radiation therapy to further improve on organ preservation strategies.

Overexpression of EGFR has been found to be significantly correlated to high tumour stage and poor patient outcome [92]. EGFR expression, however, as a predictor of prognosis, was not independent of stage. The TUXEDO trial, a phase I/II trial, is currently underway in UK, examining combination therapy of cetuximab with 5FU and mitomycin C with concurrent radiotherapy in MIBC.

Azuma et al. demonstrated in a recent trial of 134 patients receiving cystectomy or a bladder-preserving OMC/BOAI-gemcitabine regimen for locally invasive BC, a15-year OS of 40 % compared to 79.7 % in the bladder-preserving study arm [93•]. This novel form of bladder preservation therapy involves using balloon-occluded arterial infusion (BOAI) of a chemotherapeutic agent (cisplatin/gemcitabine), used concomitantly with haemodialysis and radiotherapy (known as the Osaka Medical College [OMC] regimen). This method maximises the concentration of the chemotherapeutic agent at the site of the tumour without the systemic adverse effects experienced by routine chemotherapy [94]. This method, however, can lead to the development of pelvic neuropathy. This bladder-preserving regimen can be curative in patients where both cystectomy is indicated and in those where curative treatment was previously not a possibility.

Currently the use of the tumour staging system (TNM) is insufficient to predict outcome in patients with BC irrespective of the treatment they receive. Molecular prognostic markers should be evaluated in randomised control trials in order to define their role in clinical outcome measures.

## Conclusion

Concurrent chemoradiotherapy may appear to give results equivalent to those of radical surgery with regards to local control; however, it seems to be more superior when considering quality of life. It represents an alternative for patients who decline cystectomy or where cystectomy is contraindicated. No randomised trial as of yet has directly compared bladder-preserving chemoradiotherapy with cystectomy, and therefore a definitive answer to the age-old question of superiority of one modality over other remains unanswered. One needs to understand that both organ preservation treatment and radical cystectomy are complimentary treatments rather than competing treatments. Patients opting for organ preservation need to be counselled about long-term requirement of cystoscopic surveillance and risk of losing their bladder and salvage cystectomy even decades after organ preservation therapy. The success of recent trials within the field of bladder preservation has been possible due to the meticulous selection criteria applied compared with those from previous decades. Although many chemoradiation protocols exist in the literature, few direct comparisons exist.
